# Methods in causal inference. Part 4: confounding in experiments

**DOI:** 10.1017/ehs.2024.34

**Published:** 2024-09-27

**Authors:** Joseph A. Bulbulia

**Affiliations:** Victoria University of Wellington, Wellington, New Zealand

**Keywords:** Causal inference, experiments, DAGs, evolution, per protocol effect, intention to treat effect, RCT

## Abstract

Confounding bias arises when a treatment and outcome share a common cause. In randomised controlled experiments (trials), treatment assignment is random, ostensibly eliminating confounding bias. Here, we use causal directed acyclic graphs to unveil eight structural sources of bias that nevertheless persist in these trials. This analysis highlights the crucial role of causal inference methods in the design and analysis of experiments, ensuring the validity of conclusions drawn from experimental data.

## Graphical abstract

**Figure:**
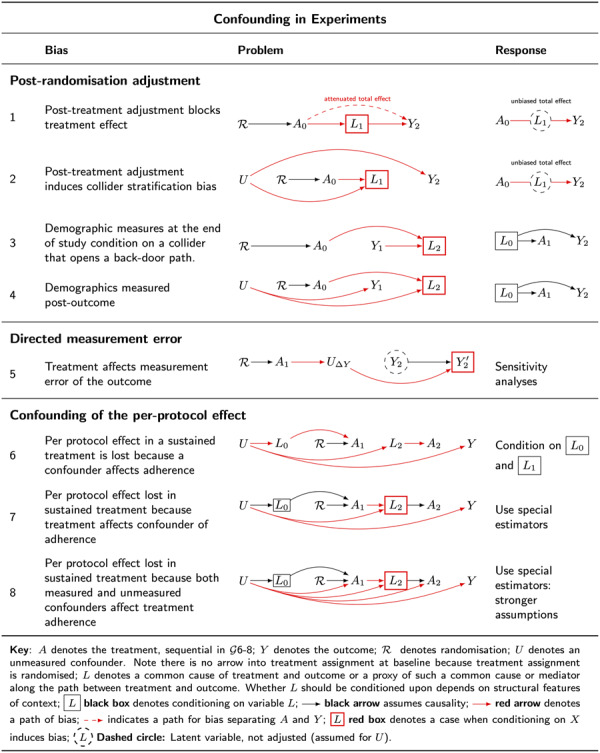

**Social media summary:** If we want to estimate causal effects, experiments are ideal. But even with successful randomisation, we only recover the effect of being randomised into treatment. Here, using causal directed acyclic graphs, we highlight common failure modes in experimental design and explain how to avoid confounding. We also show why standard methods such as ANOVA and regression may be insufficient for consistently estimating causal effects, and why special methods are sometimes needed.

## Introduction

Does not randomisation, by its very nature, eliminate all systematic causes of treatment assignment and outcome?

Yes.

Does this mean that confounding bias is ruled out?

No.

Assume large sample sizes to minimise random differences in variable distribution. Assume that the experimental trials are double-blind, with consistent treatment conditions across all arms, applied by meticulous investigators. Assume no chance event, other than randomisation. Finally, assume that the target population is not restricted in the sample population, ensuring that the experiments, if internally valid, will generalise. Assume no measurement error in the measures.

Nevertheless, biases can arise. Here, I use eight examples to illustrate common threats to valid causal inferences arising in experiments. Whereas certain risks arise from common flaws in experimental designs, such as post-randomisation selection criteria and post-randomisation covariate adjustment, hazards in estimating the ‘per-protocol effect’ of treatments do not arise from design errors. These typically require the use of methods for causal inference in ‘real world’ observational studies. The eight examples demonstrate the utility of causal directed acyclic graphs (causal DAGs) for easing the cognitive demand in diagnosing confounding bias in experimental designs. Overall, understanding how confounding occurs is crucial for experimental design, data analysis and inference, demonstrating the utility of causal inference methods for diagnosing and addressing vulnerabilities in randomised experimental designs.

We begin by defining our terms. Note that supplementary materials S1 provides a glossary of general terms used in causal inference.

### Terminology

- *Confounding bias* – treatment and outcome are associated independently of causality or are disassociated in the presence of causality relative to the causal question at hand.
- *Intention-to-treat effect (or ‘intent-to-treat effect’)* – the effect of treatment assignment, analysed based on initial treatment assignment, reflecting real-world effectiveness but possibly obscuring mechanisms.
- *Per-protocol effect* – the effect of adherence to a randomly assigned treatment if adherence were perfect (Hernán & Robins, 2017). We have no guarantee that the intention-to-treat effect will be the same as the per-protocol effect. A safe assumption is that:

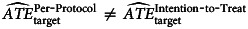

When evaluating evidence for causality, investigators should specify whether they are estimating an intention-to-treat or per-protocol effect. They should do this in addition to stating a causal contrast, effect measure and target population (Hernán, 2004; Tripepi et al., 2007) and to evaluating sources of measurement error bias (Bulbulia, 2024b).

### Meaning of symbols

We use the following conventions in our directed acyclic graphs:

- *Node* – a node or vertex represents characteristics or features of units within a population on a causal diagram, that is, a ‘variable’. In causal directed acyclic graphs, we draw nodes with respect to the *target population*, which is the population for whom investigators seek causal inferences (Suzuki et al., 2020). A time-indexed node *X_t_* denotes relative chronology; *X_ϕt_* indicates assumed timing, possibly erroneous.
- *Edge without an arrow* (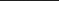) – path of association, causality not asserted.
- *Red edge without an arrow* (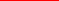) – confounding path, ignores arrows to clarify statistical dependencies.
- *Arrow* (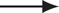) – denotes a causal relationship from the node at the base of the arrow (a parent) to the node at the tip of the arrow (a child). We typically refrain from drawing an arrow from treatment to outcome to avoid asserting a causal path from *A* to *Y* because the function of a causal directed acyclic graph is to evaluate whether causality can be identified for this path.
- *Red arrow* (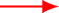) – path of non-causal association between the treatment and outcome. The path is associational and may run against arrows.
- *Dashed arrow* (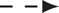) – denotes a true association between the treatment and outcome that becomes partially obscured when conditioning on a mediator, assuming *A* causes *Y*.
- *Dashed red arrow* (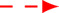) – highlights over-conditioning bias from conditioning on a mediator.
- *Boxed variable* 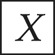 – conditioning or adjustment for *X*.
- *Red-boxed variable* 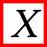 – highlights the source of confounding bias from adjustment.
- *Dashed circle* 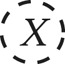 – indicates no adjustment is made for a variable (implied for unmeasured confounders).
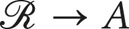
 – randomisation into a treatment condition.

### Review of d-separation for causal identification on a graph

Pearl demonstrated that causal dependencies could be evaluated by linking observable probability distributions to directed acyclic graphs (Pearl, 1995, 2009). This means that, based on assumptions about causal structure, investigators could investigate strategies for identifying causal effects from the joint distributions of observed data. The graphical rules that Pearl developed and proved are known as the rules of d-separation (Pearl, 1995), and are presented in Table 1.

**Table 1.** The five elementary structures of causality from which all causal directed acyclic graphs can be built

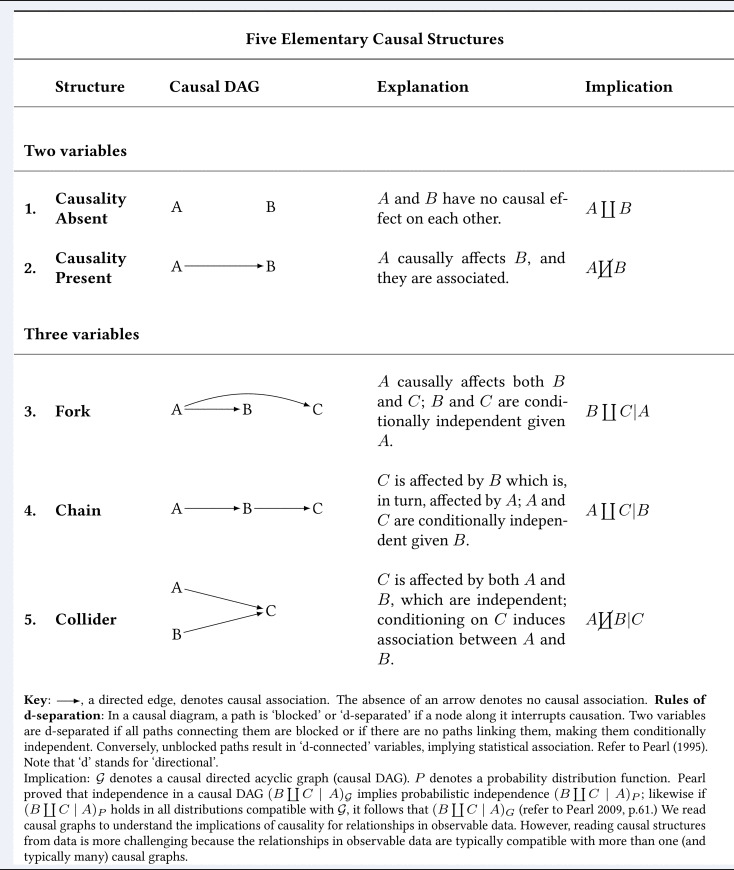

The rules of d-separation are as follows:

- *Fork rule* 
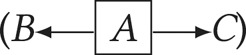
 – *B* and *C* are independent when conditioning on *A*: 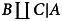.
- *Chain rule* 
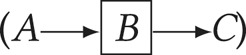
 – conditioning on *B* blocks the path between *A* and *C*: (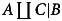).
- *Collider rule* 
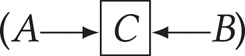
 – *A* and *B* are independent until conditioning on *C*, which introduces dependence: (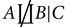).

The rules of d-separation give rise to the backdoor criterion which provides an identification algorithm conditional on the structural assumptions encoded in a causal directed acyclic graph (Pearl, 1995).

#### Backdoor adjustment

In a causal directed acyclic graph, we say that a set of variables *L* satisfies the backdoor adjustment theorem relative to the treatment *A* and the outcome *Y* if *L* blocks every path between *A* and *Y* that contains an arrow pointing into *A* (a backdoor path). Formally, *L* must satisfy two conditions:

1. *No path condition* – no element of *L* is a descendant of *A*.
2. Blocking condition – *L* blocks all backdoor paths from *A* to *Y*.

If *L* satisfies these conditions, we say that the causal effect of *A* on *Y* is identified conditional on 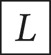. (Pearl, 2009).

## Eight examples of confounding bias in experiments

We use causal directed acyclic graphs to describe eight types of confounding bias in randomised controlled trials (‘experiments’). We use the symbol *G* to denote a causal directed acyclic graph in the table. The first digit in the graph subscript indexes the example. The second digit in the graph subscript indexes the problem or the response to the problem. Specifically, if the subscript ‘1’ is used, it refers to the graph associated with the problem; if ‘2’ is used, it refers to the graph associated with the response.

### Example 1: post-treatment adjustment blocks treatment effect

Table 2 *G*_1.1_ illustrates the threat of confounding bias by conditioning on a post-treatment mediator (McElreath, 2020). Imagine investigators are interested in whether the framing of an authority as religious or secular – ‘source framing’ – affects subjective ratings of confidence in the authority – ‘source credibility’. There are two conditions. A claim is presented from an authority. The content of the claim does not vary by condition. Participants are asked to rate the claim on a credibility scale. Next, imagine that the investigators decide they should control for religiosity. Furthermore, imagine there is a true effect of source framing. Finally, assume that the source framing not only affects source credibility but also affects religiosity. Perhaps viewing a religious authority makes religious people more religious. In this scenario, measuring religiosity following the experimental intervention will partially block the effect of the treatment on the outcome. It might make it appear that the treatment does not work for religious people, when in reality it works because it amplifies religiosity. (Note that in this graph we assume that *L_1_* occurs before *Y_2_*, however, investigators may have measured *L_1_* after *Y_2_*. Our time index pertains to the occurrence of the event, not to its measurement. This statement applies to all examples that follow.) Table 2 *G*_1.2._ clarifies a response: do not control post-treatment variables, here the intermediary effects of ‘religiosity’. If effect-modification by religiosity is scientifically interesting, measure religiosity before randomisation. Randomisation did not prevent confounding.

**Table 2.** Eight confounding biases in Randomised Controlled Trials

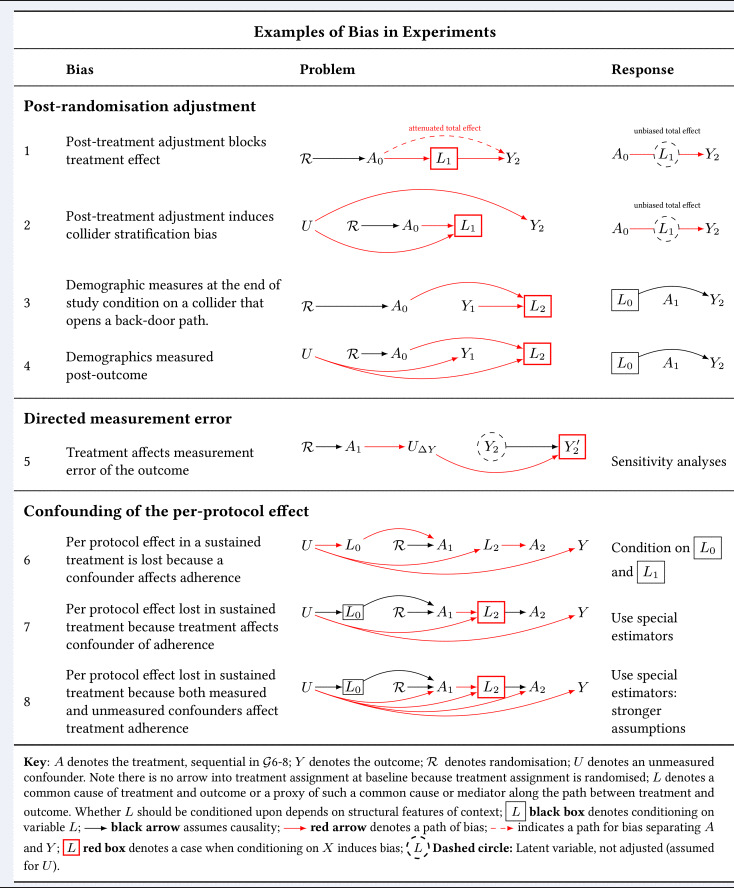

### Example 2: post-treatment adjustment induces collider stratification bias

Table 2 *G*_2.1_ illustrates the threat of confounding bias by conditioning on a post-treatment collider (Cole et al., 2010). Imagine the same experiment as in Example 1 and the same conditioning strategy, where religiosity is measured following the treatment. We assume that the treatment affects religiosity. However, in this example, religiosity has no causal effect on the outcome, source credibility. Finally, imagine an unmeasured variable affects both the mediator, religiosity (*L*_1_), and the outcome, source credibility (*Y*_2_). This unmeasured confounder might be religious education in childhood. In this scenario, conditioning on the post-treatment variable religiosity will open a backdoor path between the treatment and outcome, leading to an association in the absence of causation. Randomisation did not prevent confounding.

Table 2 *G*_2.2_ clarifies a response: do not control post-treatment variables. The point that investigators should not condition on post-treatment variables can be illustrated with a common flaw in experimental designs: exclusion based on ‘attention checks’. Consider that if an experimental condition affects attention and an unmeasured variable is a common cause of attention and the outcome, then selection on attention will induce confounding bias in a randomised experiment. For example, imagine that people are more attentive to the scientific authority design because science is interesting – whether or not one is religious, yet religion is not interesting whether or not one is religious. Suppose further that an unmeasured ‘altruistic disposition’ affects both attention and ratings of source credibility. By selecting on attention, investigators may unwittingly destroy randomisation. If attention is a scientifically interesting effect modifier, it should be measured before random assignment to treatment.

### Example 3: demographic measures at end of study induce collider stratification bias

Table 2 *G*_3.1_ illustrates the threat of confounding bias from adjusting for post-treatment variables, here, one affected by the treatment and outcome absent any unmeasured confounder. In our example, imagine that both the treatment, source framing, and the outcome, source credibility, affect religiosity measured at the end of the study. Investigators measure religiosity at the end of the study and include this measure as a covariate. However, doing so induces collider bias such that if both the treatment and outcome are positively associated with religiosity, the collider, they will be negatively associated with each other. Conditioning on the collider risks the illusion of a negative experimental effect without causality.

Table 2 *G*_3.2_ clarifies a response: again, do not control post-treatment variables. Here, ‘religiosity’ is measured after the end of the study. If the scientific interest is in effect modification or obtaining statistical precision, measure covariates before randomisation.

### Example 4: demographic measures at end of study condition on a collider that opens a backdoor path

Table 2 *G*_4.1_ illustrates the threat of confounding bias by adjusting for post-treatment variables, here affected only by the treatment and an unmeasured cause of the outcome. Suppose source credibility affects religiosity (religious people are reminded of their faith), but there is no experimental effect of framing on credibility. Imagine further that there is an unmeasured common cause of the covariate religiosity and the outcome source credibility. This unmeasured confounder might be religious education in childhood. In this scenario, conditioning on the post-treatment variable religiosity will open a backdoor path between the treatment and outcome, leading to an association without causation. Again, we find that randomisation did not prevent confounding.

Table 2 *G*_4.2_ clarifies a response. Again, unless investigators can rule out an effect of treatment, they should not condition on a post-treatment covariate. The covariates of interest should be measured before randomisation.

### Example 5: treatment affects attrition biasing measure of outcome

Table 2 *G*_5_ – suppose that the experimental condition affects measurement error in self-reported source credibility *U*_Δ*Y*_. For example, suppose that source framing has no effect on credibility. However, those in the scientific authority condition are more likely to express credibility for science owing to self-presentation bias. Likewise, perceiving the investigators to be irreligious, participants in the religious source framing condition might report less credibility for religious authorities than they secretly harbour. Directed measurement error from the treatment to the measurement error of the outcomes creates an association without true causality, which we denote by removing any arrow between the treatment *A* and the true outcome *Y*. Note that the bias in this setting is not one of confounding bias. There is no common cause of treatment and outcome. Rather, the threat is from measurement error bias (refer to Bulbulia, 2024b).

Table 2 *G*_5_ suggests there is no easy solution to directed measurement error bias in this setting. If the magnitude of the measurement error bias were known, investigators could apply adjustments (Lash et al., 2009). Additional experiments might be devised that are less prone to directed measurement error bias. Investigators might compute sensitivity analyses to examine how much measurement error bias would be required to explain away a result (refer to Linden et al., 2020 for a relatively easy-to-implement sensitivity analysis). The point we make here is that randomisation does not prevent bias arising from directed measurement error. Investigators must be vigilant.

### Example 6: per protocol effect lost in sustained treatments where treatment adherence is affected by a measured confounder

Setting aside self-inflicted injuries of post-treatment conditioning and directed measurement error, *randomisation recovers unbiased causal effect estimates for randomisation into treatment*. Under perfect adherence, such estimates correspond to the causal effects of the treatments themselves. However, adherence is seldom perfect. The following examples reveal challenges for recovering per-protocol effects in settings where there is imperfect adherence. Table Table 2 *G*_6–8_ are adapted from Hernán and Robins (2017).

Table 2 *G*_6_ illustrates the threat for identifying the per-protocol effect in sustained treatments where treatment adherence is affected by a measured confounder. Consider a sequential experiment that investigates the effects of sustained adherence to yoga on psychological distress, measured at the end of the study. Suppose that inflexible people are less likely to adhere to the protocols set out in the experiment and therefore do not. Suppose that flexibility is measured by indicator *L*. If we do not condition on *L*, there is an open path from, 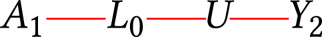. Although investigators may recover the effect of randomisation into treatment, the per-protocol effect is confounded.

Table 2 *G*_6_ also clarifies a response. Conditioning on *L*_0_ and *L*_1_ will block the backdoor path, leading to an unbiased per-protocol effect estimate.

### Example 7: per protocol effect lost in sustained treatments where past treatments affect measured confounder of future treatment adherence

Table 2 *G*_7_ illustrates the threat for identifying the per-protocol effect in sustained treatments where past treatments affect measured confounder of future treatment adherence. Suppose that yoga affects flexibility. We should condition on pre-treatment measures of flexibility to identify the per-protocol effect. However, conditioning on the post-treatment measure of flexibility, 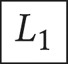 induces collider stratification bias. This path runs from 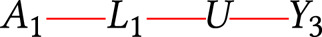. However, if we do not condition on *L*_1_ there is an open backdoor path from 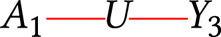. We cannot estimate a per-protocol effect by conditioning strategies.

Table 2 *G*_7_ suggests no easy remedy for obtaining valid causal inference in this setting. However, in a sequential treatment with fixed strategies, in which there is sequential exchangeability – or no unmeasured confounding at each time point – valid estimators for the sequential treatments may be constructed (refer to Hernán & Robins, 2024; Díaz et al., 2021); Hoffman et al., 2023). Although we may naively obtain an intention-to-treat effect estimate without special methods, inferring an effect of doing yoga on well-being – the per-protocol effect, requires special methods. Such methods are not yet routinely used in the human sciences (Bulbulia, 2024a).

### Example 8: per protocol effect lost in sustained treatments because both measured and unmeasured confounders affect treatment adherence

Table 2 *G*_8_ illustrates the threat for identifying the per-protocol effect in sustained treatments with measured and unmeasured confounders. Suppose flexibility affects adherence, yoga affects flexibility and an unmeasured variable, such as prejudice towards Eastern spiritual practices, affects adherence. We have no measures for this variable. There is unmeasured confounding.

If there were no effect of yoga on well-being except through flexibility, and furthermore if flexibility were not affected by the unmeasured antipathy towards Eastern spiritual practices, and further, if the effect of flexibility on yoga at each time point were conditionally independent of all future counterfactual data, both for the treatments and the outcomes, then it might be possible to construct special estimators that identify the per-protocol effect of yoga on well-being in the presence of unmeasured confounding that affects adherence (refer to Hernán & Robins, 2017). These special estimators are quite different from the ANOVAs, regressions models and multi-level regression models routinely deployed in experimental studies. However, if we seek to understand the effect of yoga on well-being and not the effect of random assignment to yoga on well-being the routine estimators will not work: we require special estimators (Díaz et al., 2023; Hernán & Robins, 2024; Hoffman et al., 2023).

## Summary

The examples considered here do not exhaust all threats to causal inference in experiments. For example, I have not covered biases arising from sample attrition also known as right censoring bias (refer to Bulbulia, 2024b). However, I hope the eight examples presented persuade experimental investigators of the following.

First, there is no need to adjust for baseline confounders in a non-sequential randomised experiment. Although an unadjusted difference of means should be reported, Lin has shown that if a study is sufficiently powered, regression adjustment where the full set of treatments are interacted with baseline covariates may improve (and will not diminish) asymptotic precision (Lin, 2013). In some settings, investigators will want to evaluate effect modification with strata of covariates at baseline. Regression with an interaction term is only sufficient to identify effect modification under the assumption of constant linear effects across all strata to be compared. This assumption is typically implausible. Recent developments in non-parametric estimation offer hope, though these methods have yet to be fully tested and require large sample sizes (Wager & Athey, 2018). In short, even fundamental questions such as for whom treatment effects apply remain elusive. Nevertheless, with sufficiently large samples, randomisation ensures balance in confounders, allowing, if all else goes well, the recovery of average treatment effects for the study population.

Second, confounding biases can occur in randomised experiments even when randomisation succeeds. To evaluate such bias, we must first state whether our causal estimand is the intention-to-treat effect or the per-protocol effect. Randomisation recovers an unbiased estimate of the intention-to-treat effect – that is, the effect of treatment assignment. Randomisation will only recover the per-protocol effect, the effect of following treatment, when those assigned to treatment adhere to their assignments.

Third, causal directed acyclic graphs are useful for clarifying sources of bias for both the intention-to-treat effect and the per-protocol effect. For the intention-to-treat effect, biases arise in two main ways: when investigators impose selection criteria on participants after randomisation (e.g. assessing treatment effects only in those who have followed protocols) or when investigators estimate treatment effects using covariates collected after randomisation. Both post-treatment selection and post-treatment conditioning are self-inflicted sources of confounding bias. The remedy is to not allow your design to compromise randomisation. For the per-protocol effect, randomisation cannot guarantee unbiased estimates. Obtaining consistent estimates for the per-protocol effect requires the same assumptions and methods that are required when estimating causal effects in observational studies.

Fourth, in a sequential randomised experiment, standard methods such as regression adjustment, statistical structural equation models and multi-level models will often fail to yield unbiased estimands (Bulbulia, 2024a; Richardson & Robins, 2013; Young et al., 2014). Special estimators such as ‘g-methods’ (Hernán & Robins, 2024) or targeted learning (Van Der Laan & Rose, 2018) may be necessary to recover per-protocol effects in sequential designs. The requirements for estimating per-protocol effects in experiments cannot be stated in isolation from the details of each study (Hernán & Robins, 2024; Robins, 1986).

From these observations, we offer the following practical advice:

1. Ensure covariate data are collected before randomisation into treatments.
2. If attention is a relevant covariate, measure it before randomisation. Do not use ‘attention checks’ to select participants after randomisation into treatments.
3. If adjustment is used in a single-point treatment with baseline covariates, interact every level of treatment with the baseline covariates, following Lin (2013), recognising that interaction coefficients cannot be interpreted as evidence of multiplicative effect modification (moderation) except under strong assumptions of constant linear interaction effects and common support for these effects in the data (Hainmueller et al., 2019).
4. For sequential treatments, collect data for adherence (where possible).
5. For sequential treatments, at each measurement interval, ensure covariate data collection for any variable that might affect adherence or that might be proxies for such variables, particularly if these variables, or proxies for these variables, might affect outcomes at the end of the study.
6. Do not infer per-protocol effects from the portion of the sample that followed experimental protocols. Such selection can lead to differences between the study population at the start and end, compromising external validity.
7. Where possible, report both the per-protocol effect and the intention-to-treat effect.

Describing the specialised methods for estimating per-protocol effects with multiple sequential treatments is beyond the scope of this commentary (Hernán & Robins, 2024). My aim has been to show that meeting the assumptions for valid causal inferences in experiments is often more challenging than many experimental human scientists currently appreciate (refer to Montgomery et al., 2018). This is not meant as a criticism of others—my own work is not exempt. For example, I was part of an international team that administered questionnaires on religious identification after randomisation in a cross-cultural study investigating the source credibility of religious and scientific authority (Hoogeveen et al., 2022). This approach may have diminished the effect modification by religiosity. Causal inference methods hold significant potential to improve the design, analysis, and interpretation of experimental research, ultimately advancing everyone's contributions, including my own.

## Supporting information

Bulbulia supplementary material Bulbulia supplementary material

## Research transparency and reproducibility

No data were used in this manuscript.
