# Supplementary material for: Methods in causal inference. Part 4: confounding in experiments
Source: Evol Hum Sci. 2024 Sep 27;6:e43. doi: 10.1017/ehs.2024.34 (PMC11658928; doi:10.1017/ehs.2024.34)
Supplement: Bulbulia supplementary material [file S2513843X24000343sup001.pdf]

Supplementary files for “Methods in Causal Inference Part 4: Confounding in Experiments”

Joseph A. Bulbulia[1]

[1] Victoria University of Wellington, New Zealand ORCID 0000-0002-5861-2056

2024-06-20

Table of contents

S1. Glossary 2

List of Tables

1 Glossary 3

S1. Glossary

Table 1: Glossary

Term	Definition
**Acyclic**	No variable can be an ancestor or descendant of itself on a causal graph.
**Adjacent Nodes**	Two nodes connected by an arrow are adjacent.
**Adjustment Set**	Variables conditioned to block all backdoor paths between treatment (A) and outcome (Y).
**Ancestor/Descendants**	Nodes connected by directed edges. All descendants of an ancestor can be reached by directed paths.
**Arrow**	Represents direct causation in a causal diagram, pointing from cause to effect.
**Average Treatment Effect (ATE)**	The difference in expected outcomes between treated and untreated units across a specified population. Synonym for Marginal Effect.
**Backdoor Path**	Path that, if not blocked, may associate the treatment and outcome without causality.
**Causal Contrast**	The difference in expected outcomes under different treatment levels.
**Causal Contrast Scale**	The metric for quantifying causal contrasts, chosen based on outcome type and research question.
**Causal Diagram (Causal DAG)**	A graph representing causal relationships to evaluate an identification problem; must be acyclic and describe all confounding, measured and unmeasured for the target population.
**Causal Estimand**	The causal contrast of interest in a study; specifies the intervention, outcome, contrast scale, and target population; stated before analysis.
**Causal Path**	Asserts a change in the parent node will induce a change in its child.
**Censoring**	the sample population is not representative of the target population at baseline (left censoring) or is no longer representative at the end of study (right censoring).
**Collider/"Immorality"**	A variable where two causal paths meet head-to-head, may induce non-causal associations between its parents.
**Conditional Average Treatment Effect (CATE)**	The treatment effect for specific subgroups, defined by measured characteristics.
**Conditioning**	Adjustment for variables in analysis to distinguish causal effects from associations.
**Confounding**	Treatment and outcome are associated independently of causality or are disassociated despite causality, relative to the causal question.
**Confounder**	A variable or set of variables form part of an ideal identification strategy to reduce or eliminate confounding.
**Counterfactual or Potential outcomes**	Hypothetical outcomes under different treatment conditions to be contrasted, only one may be realised for each observed unit.
**Direct Effect (Natural Direct Effect)**	The difference between potential outcomes when the treatment is applied and the mediator is set to no-treatment versus when neither the treatment nor the mediator is applied.
***d*-separation**	Backdoor paths are blocked, satisfying the assumption of 'no unmeasured confounding'.
**Descendant (Child)**	A node causally influenced by a prior node (Parent). A child is a parent's direct descendant.
**Effect-Measure Modifier/Effect-Modifier**	A variable that affects the magnitude or direction of a causal effect.
**Estimator**	Algorithm to compute a statistical estimand from data.
**External Validity/Target Validity**	The generalisability of study findings to the prespecified target population; assumes internal validity.
**Factorisation**	Decomposing the joint probability distribution of variables into a product of conditional probabilities of each variable given its parents.
**Heterogeneous Treatment Effects**	Variation in treatment effects across subgroups or contexts.
**Identification Problem**	Ensure no unmeasured confounding.
**Incident Exposure Effect**	Causal effect of initiating a new treatment.
**Indirect Effect (Natural Indirect Effect)**	The average difference in potential outcomes when the mediator is at its natural value under treatment versus no treatment.
**Instrumental Variable**	Associated with treatment but affecting the outcome only through the treatment, used for estimating causal effects amidst confounding.
**Intention-to-Treat Effect**	The effect of treatment assignment, what random assignment obtains.
**Internal Validity**	The extent to which causal associations in the study population are accurately identified.
**Inverse Probability of Censoring Weights**	Weights used to adjust for bias due to attrition in longitudinal studies.
**Inverse Probability of Treatment Weights**	Weights that create a pseudo-population to achieve treatment balance across conditions.
**Local Markov Assumption**	assumption that a variable is independent of its non-descendants given its immediate parents in a causal graph.
**Longitudinal Study/Panel Study**	A research design that repeatedly tracks and measures the same units over time.
**Loss-to-follow-up**	Participant attrition.
**Markov Assumption**	assumption that a variable is independent of its non-descendants given its parents in a causal graph
**Marginal Effect**	Synonym for Average Treatment Effect.
**Measurement Error Bias**	Bias introduced when measurements of variables are inaccurately recorded, either through correlated or direct measurement errors, or when uncorrelated errors mask the true effects.
**Mediator**	A variable through which a treatment affects an outcome.
**Modularity Assumption**	Interventions on one set of variables do not directly alter the conditional distribution of other variables, given their direct causes.
**Node**	Represents a variable in a causal diagram, also called "Vertex"
**Observational Study**	Treatment assignment is not controlled by the investigator.
**Parent/Child**	Adjacent nodes connected by a directed path.
**Path**	Nodes are connected by a sequence of edges. Directed paths follow directed edges.
**Per-Protocol Effect**	The causal effect under full-treatment adherence.
**Prevalent Exposure Effect**	Effect of current or ongoing treatments.
**Propensity Score**	The probability of receiving a treatment based on observed characteristics used for confounding adjustment in observational studies.
**Randomised Treatment Assignment**	Chance treatment assignment.
**Randomised Controlled Trial (RCT)**	Uses random treatment assignment to balance confounders across the treatments to be compared.
**Reverse Causation**	Mistaking the effect for the cause in an analysis.
**Sample Weights**	Adjusts sample data to represent the target population in analysis better.
**Selection Bias**	Systematic errors from non-representative study participation or attrition affecting generalisability.
**Sequentially Treatment**	multiple treatments may be fixed our time-varying
**Single World Intervention Graph (SWIG)**	A graph to obtain causal identification under a single counterfactual treatment regime by splitting nodes into random and fixed components, where the fixed inherits edges directed into the node (parents) and the random inherits edges out (children).
**Single World Intervention Template (SWIT)**	A graph-valued function or template generates SWIGs (is not itself a graph).
**Statistical Estimand**	The parameter of interest in a statistical model, not necessarily causal.
**Statistical Estimate**	The value obtained for a statistical estimand from data analysis.
**Statistical Model**	Describes covariance between variables; without structural assumptions, statistical models do not identify causal effects.
**Structural Model**	Assumptions about causal relationships encoded in diagrams, essential for identifying causality from statistical associations.
**Study Population**	The population from which data are collected, also called the "sample population."
**Target Population**	The broader population to which study results are intended to apply.
**Target Trial**	An observational study emulating an ideal experiment by pre-specifying a causal estimand, eligibility criteria, and data ordering for an incident exposure effect.
**Time-Varying Confounding**	Confounding that changes over time, complicating causal effect estimation using standard methods.
**Total Effect**	The difference in mean potential outcomes under contrasted treatments in a study.
